# Optimization of Xylanase Production through Response Surface Methodology by *Fusarium* sp. BVKT R2 Isolated from Forest Soil and Its Application in Saccharification

**DOI:** 10.3389/fmicb.2016.01450

**Published:** 2016-09-22

**Authors:** Golla Ramanjaneyulu, Bontha Rajasekhar Reddy

**Affiliations:** Department of Microbiology, Sri Krishnadevaraya UniversityAnantapuramu, India

**Keywords:** *Fusarium* sp., optimization, response surface methodology, saccharification, submerged fermentation, xylanase

## Abstract

Xylanses are hydrolytic enzymes with wide applications in several industries like biofuels, paper and pulp, deinking, food, and feed. The present study was aimed at hitting at high yield xylanase producing fungi from natural resources. Two highest xylanase producing fungal isolates—Q12 and L1 were picked from collection of 450 fungal cultures for the utilization of xylan. These fungal isolates—Q12 and L1 were identified basing on ITS gene sequencing analysis as *Fusarium* sp. BVKT R2 (KT119615) and *Fusarium strain* BRR R6 (KT119619), respectively with construction of phylogenetic trees. *Fusarium* sp. BVKT R2 was further optimized for maximum xylanase production and the interaction effects between variables on production of xylanase were studied through response surface methodology. The optimal conditions for maximal production of xylanase were sorbitol 1.5%, yeast extract 1.5%, pH of 5.0, Temperature of 32.5°C, and agitation of 175 rpm. Under optimal conditions, the yields of xylanase production by *Fusarium* sp. BVKT R2 was as high as 4560 U/ml in SmF. Incubation of different lignocellulosic biomasses with crude enzyme of *Fusarium* sp. BVKT R2 at 37°C for 72 h could achieve about 45% saccharification. The results suggest that *Fusarium* sp. BVKT R2 has potential applications in saccharification process of biomass.

## Introduction

Lignocellulosic materials are exclusively generated as energy crops and waste/by products derived from the several agro-industries like sugarcane processing industries, food processing industries, forestry industries (Womersley, 2006; Candido et al., 2012). Agriculture and Municipalities contribute considerable proportions of feedstock of lignocellulosic solids for large scale industrial fermentation for conversion to value added end-products. Lignocellulose is the primary building block of plant cell walls of woody and non-woody plants and a renewable natural resource through photosynthesis. The chemical properties of components of lignocellulosics make them a substrate of enormous biotechnological value. The composition of lignocellulosic biomass varied with mainly cellulose (35–50%), followed by hemicellulose (20–35%) and lignin (10–25%; Sun and Cheng, 2002).

Hemicellulose mainly constitutes xylan backbone of xylose residues linked by β-1, 4-glycosidic bonds (Beg et al., 2001). Softwood and hardwood xylans are similar in the sense that both the xylans have a reducing end group mainly consisting of rhamnosyl, galactosyl, acetyl, arabinosyl, glucuronosyl, or xylosyl residue.

Microflora in different niches plays a pivotal role in cycling of lignocellulosic mass with considerable proportion of xylan in biogeochemical cycles (Wall and Virginia, 1999; Wardle, 2002; Hackl et al., 2004; Kirk et al., 2004; Nandhini and Josephine, 2013). Understanding of population of xylan—utilizing microflora in different ecosystems is a basic step for exploration of xylanolytic organisms. Xylanolytic organisms, inhabitants of certain ecological niches, have capability to secrete xylanases (endo-1, 4-β-D-xylanohydrolase; EC 3.2.1.8) for utilization of xylan in plant cell wall (Sridevi and Charya, 2011; Singh et al., 2013; Kulkarni and Gupta, 2013; Kumar et al., 2014; Ramanjaneyulu et al., in press). Though xylanases have been detected in certain bacteria and filamentous fungi (Beg et al., 2001), the vast diversity of untapped fungal species inhabiting niches still exists and needs to be explored for production of xylanase (Lu et al., 2008). In view of utility microbial xylanases in different biotechnological applications in various processes—biofuel production, preparation of animal feed and bleaching of pulp and paper, the pharmaceutical, food, and feed industries, clarification of fruit juices and degumming of vegetal fibers, production of xylanase in large scale by fermentation methods is gaining more importance and there is a lot of scope in cutting down cost of enzyme with use of high yielding cultures. Keeping this into consideration, the present study was aimed at screening of fungal cultures isolated from different regions of forest in Eastern Ghats of Andhra Pradesh, India for production of xylanase and optimization of xylanase by the potential fungal culture and assessment of saccharifying ability of crude enzyme of the potential organism.

## Materials and methods

### Culture medium

Mineral salts medium (MSM), used in the present study contained the following ingredients—NH_4_NO_3_: 1.5 g; KH_2_PO_4_: 2.5 g; NaCl: 1.0 g; MgSO_4_: 1.5 g; MnSO_4_: 0.01 g; FeSO_4_: 0.005 g; CaCl_2_: 0.05 g; xylan: 1.0 g; and Agar Agar: 20 g dissolved in 1000 ml of distilled water. pH of the medium was adjusted to 5.0 and sterilized at 121°C and 15 lb of pressure for 20 min and poured into sterile petri dishes. The same solid medium and liquid medium devoid of agar were also used for the maintenance of cultures and production of xylanase by the fungal cultures, respectively.

### Fungal cultures

There are about 450 fungal cultures in our stock which were isolated on MSM plates by serial dilution method from soil samples collected from different locations of Eastern Ghats of Andhra Pradesh, India by Ramanjaneyulu et al. (in press) and maintained on MSM. Fifteen promising cultures from this collection were selected for the present study through primary screening on the basis of colorless zone formation in plates for xylanase production (Ramanjaneyulu et al., 2015). Fungal isolates were given code numbers according to location of soil samples used for isolation.

### Production of xylanase in submerged fermentation (SmF)

Xylanase production by the 15 fungal cultures was assessed in submerged fermentation in liquid MSM medium with 0.1% birchwood xylan as the sole carbon source. Thirty milliliters of liquid MSM in 250 ml Erlenmeyer flasks were inoculated with five 0.5 mm agar plugs of 5 days old culture and incubated at 30°C and 150 rpm in an incubator cum shaker (Remi 24BL) for 7 days. The samples were withdrawn at specific time interval and filtered through Whatman No. 1 filter paper and the filtrate was centrifuged at 8000 g (Remi C–24) at 4°C for 10 min. The supernatant was collected and used as an enzyme source for the assay of xylanase activity and cellulase activity. The isolate showing higher production of xylanase under submerged fermentation were further optimized for various physical and chemical parameters.

### Xylanase assay

Xylanase assay was based on measurement of xylose sugar released from xylan substrate according to the method of Bailey et al. (1992). Supernatant of culture of fungal isolates in different experiments at different intervals was used as a source of enzyme because of extracellular nature of the enzyme. Xylanase assay involved initiation of reaction in assay mixture containing 1.0 ml of crude enzyme source, 1.0 ml of 1% birch wood xylan in Na-citrate buffer (0.05 M, pH 5.3) and 1.0 ml of citrate buffer upon incubation at 55°C for 10 min followed by termination with addition of 3.0 ml of 3, 5-dinitrosalisylic acid (DNS) (Miller, 1959). An amount of xylanase enzyme releasing 1 μmol of xylose from xylan in 1 min is considered as one unit (U) of xylanase activity.

### Carboxymethyl cellulase (CMCase) assay

Supernatant derived from culture of fungal isolates was also tested for carboxymethyl cellulase activity and used as a source of enzyme. Assay of CMCase activity relied on incubation of enzyme source with Carboxy methyl cellulose (CMC) substrate in Na-acetate buffer (0.05 M, pH 5.3) in a final volume of 3 ml at 50°C for 15 min (Casimir-Schenkel et al., 1996). The reaction was stopped by the addition of 3.0 ml of DNS and the contents were boiled for 15 min in water bath (Miller, 1959). The color developed was read at 540 nm. The amount of reducing sugar liberated was quantified using glucose as standard. One unit of cellulase activity is defined as the amount of enzyme that liberates 1 μmol of glucose equivalents per min under the assay conditions (Mandels et al., 1981).

### Protein estimation

Content of the soluble protein in the culture filtrate of fungal isolates grown in different experiments were determined according to Folin's method using bovine serum albumin as standard (Lowry et al., 1951).

### Biomass measurement

Mycelial mat and culture filtrate were separated by passing culture broth of fungal isolates from different experiments through Whatman filter paper No. 1. Dry weight of mycelial mat recovered on filter paper after drying at 70°C in an oven represented biomass in mg/flask.

### Molecular identification and phylogenetic analysis

Identification of the two potential fungal isolates with high titers of xylanase was relied on base sequence of Internal Transcribed Spacer (ITS) of ribosomal DNA (rDNA) genes of the potential culture. For this purpose, total genomic DNA was extracted from cultures of the selected fungal isolates with QIAamp DNA Mini Kit, (Quiagen, Germany) following the method of Sambrook et al. (1989) according to instructions of manufacturer. Internal Transcribed Spacer-1(ITS-1) region of rDNA was amplified on the purified genomic DNA of the fungal isolates by using a set of two oligonucleotide fungal primers of forward primer ITS-5F 5′-GGAAGTAAAAGTCGTAACAAGG-3′ and reverse primer ITS-4R 5′-TCCTCCGCTTATTGATATGC-3′ in PCR reaction.

All PCR reagents in PCR reaction mixture were from Merck (Bangalore Genei). PCR reaction was performed (Applied Biosystems GeneAmp PCR System 9700, USA) in reaction mixture in a total volume of 25 μl in an eppendorf containing DNA template (1.5 μl), 10X Reaction buffer (2.5 μl), forward and reverse primers (ITS-5F and ITS-4R) (0.5 μl), dNTP mixture (0.5 μl), Taq polymerase (0.3 μl), BSA (0.3 μl), and Milli Q water (18.9 μl). Initial denaturation was carried out at 94°C for 5 min, the samples were run for 40 cycles with the following temperature profile; melt temperature 94°C for 30 s, annealing temperature 62.5°C for 30 s, extension temperature of 72°C for 45 s, final extension temperature 72°C for 7 min. After the completion of PCR the reaction mixture was checked for bands in 1% agarose gel electrophoresis. PCR products were sequenced using ABI PRISM Big Dye Terminator cycle sequencing ready reaction kit.

### Analysis of sequence data

The raw sequences (forward and reverse) were assembled with a reference sequence using DNA Baser software. These assembled contigs were searched for similarities by using NCBI-BLAST for the Gene Bank database. Selected sequences with the greatest similarity were collected and compared for phylogenetic relationship (Dereeper et al., 2008) in molecular evolutionary analysis 6.0v (MEGA. 6.0v) software and further analysis was carried out to generate phylogenetic tree by Neighbor-joining method.

### Optimization of xylanase production by response surface methodology (RSM)

Initially, batch experiments in 250 ml-Erlenmeyer flasks with 30 ml of MSM were conducted with One Factor at a Time (OFAT) approach for selection of important variables affecting xylanase production by the promising *Fusarium* sp. BVRK2 in the same manner as mentioned earlier. A total of 50 batch experiments in central composite design (CCD; Design expert, Stat-Ease) through RSM with the five selected independent variables—Sorbitol, Yeast extract, different initial temperatures, pH, and agitation at different levels with coded values as shown in Table 1 and at different levels as indicated in Table 2 were performed to find out interaction effects between variables for maximization of xylanase production. Quantitative data generated from these experiments were subjected to analysis of regression through response surface methodology to solve multivariate equations. In performing regression equation, response values obtained under influence of variable at high (+1) and low (−1) levels were coded according to the following equation

$$\begin{array}{l} x_{i}=\frac{X_{i}-X_{cp}}{{\delta X}_{i}} \end{array}$$

in which *x*_*i*_ is the coded value of an independent variable, *X*_*i*_ is the real value of an independent variable, *X*_*cp*_ is the real value of an independent variable at the center point, and δ*X*_*i*_ is the step change value.

**Table 1:** **Range and levels of independent variables for the central composite design used in xylanase production**.

**S. No**.	**Variables**		**Level**	
	**Parameter code**	**Parameter**	**Low (−1)**	**High (+1)**
1	A	Sorbitol (%)	0.5	2.5
2	B	Yeast extract (%)	0.5	2.5
3	C	pH	3.0	7
4	D	Temperature (°C)	20	45
5	E	Agitation (rpm)	100	250

**Table 2:** **Central composite design (CCD) matrix of factors in coded values along with enzyme activity as response**.

**Run**	**Block**	**Sorbitol**	**Yeast extract**	**pH**	**Temperature**	**Agitation**	**Actual**	**Predicted**
	**1**	**(%) (A)**	**(%) (B)**	**(C)**	**(°C) (D)**	**(rpm) (E)**	**(U/ml)**	**(U/ml)**
1	{1}	−1	−1	1	1	1	345	323.23
2	{1}	0	0	0	0	0	4560	4723.53
3	{1}	1	1	1	1	1	1295	1284.07
4	{1}	1	−1	1	−1	1	1275	1249.84
5	{1}	0	1	0	0	0	4485	4523.39
6	{1}	0	0	0	0	1	4545	4353.68
7	{1}	−1	−1	−1	−1	1	385	368.59
8	{1}	0	0	−1	0	0	4525	4301.04
9	{1}	1	−1	−1	−1	−1	1295	1303.92
10	{1}	1	−1	1	−1	−1	1398	1424.29
11	{1}	0	0	0	0	0	4450	4723.53
12	{1}	1	1	−1	−1	−1	1350	1352.64
13	{1}	0	0	0	0	0	4450	4723.53
14	{1}	−1	−1	1	−1	1	400	375.21
15	{1}	−1	−1	−1	1	1	425	405.98
16	{1}	0	0	0	0	0	4450	4723.53
17	{1}	−1	1	1	−1	1	435	478.3
18	{1}	0	−1	0	0	0	3490	2480.16
19	{1}	−1	1	1	1	1	320	395.69
20	{1}	1	−1	−1	−1	1	1300	1320.1
21	{1}	0	0	1	0	0	4540	4335.16
22	{1}	−1	−1	1	−1	−1	395	377.78
23	{1}	0	0	0	1	0	4545	4323.98
24	{1}	−1	1	1	1	−1	320	299.51
25	{1}	1	−1	−1	1	1	1250	1301.87
26	{1}	0	0	0	0	0	4560	4713.53
27	{1}	−1	1	−1	−1	−1	415	429.88
28	{1}	1	1	−1	1	−1	495	478.15
29	{1}	−1	0	0	0	0	2495	2480.16
30	{1}	0	0	0	−1	0	4595	4387.21
31	{1}	1	1	−1	1	1	1350	1343.08
32	{1}	−1	1	−1	−1	1	410	471.06
33	{1}	0	0	0	0	−1	4545	4307.57
34	{1}	1	−1	−1	1	−1	1260	1280.06
35	{1}	−1	−1	1	1	−1	410	370.17
36	{1}	−1	1	−1	1	−1	415	461.02
37	{1}	1	1	1	−1	1	1350	1372.31
38	{1}	−1	1	1	−1	−1	320	307.75
39	{1}	−1	−1	−1	1	−1	440	382.21
40	{1}	−1	−1	−1	−1	−1	435	420.54
41	{1}	1	1	1	−1	−1	1345	1343.63
42	{1}	1	1	1	1	−1	1195	1189.77
43	{1}	1	−1	1	1	1	1360	1392.23
44	{1}	0	0	0	0	0	4555	4723.53
45	{1}	1	1	−1	−1	1	1230	1291.94
46	{1}	1	0	0	0	0	4340	4261.04
47	{1}	1	−1	1	1	−1	1345	1341.06
48	{1}	0	0	0	0	0	4500	4723.53
49	{1}	0	0	0	0	0	4548	4723.53
50	{1}	−1	1	−1	1	1	430	427.82

### Statistical analysis

Experimental data were analyzed with a statistical software package Design Expert (Stat-Ease, Inc Minnepolis, MN) to find out relationship between variables and the yields of xylanase enzyme through quadratic equation. Fitness of data into equation has been validated with coefficient of variation (*R*^2^) in statistical test called analysis of variance (ANOVA) along with statistical significance as determined by *F*-test.

### Saccharification of biomass

Untreated plant biomass—sawdust, rice straw and cotton stocks were collected, chopped and sieved through 1.0 mm sieve and used for saccharification. The saccharification was carried out in 250 ml Erlenmeyer flask at 5% solid loading in citrate buffer (0.05 M) at pH of 5.0 with enzyme (crude enzyme) loading of 50 U/g of biomass. The culture filtrate, used as a source of crude enzyme for saccharification, contained 4560 U/ml with specific activity of 797 U/mg of protein. To inhibit microbial contamination during saccharification, 0.01% sodium azide was used and incubated at 150 rpm in orbital shaker at 37°C and the flasks were withdrawn at specific time intervals filtered through Whatman No. 1 filter paper and the filtrate were centrifuged at 12,000 g for 10 min. Clear supernatant was used to estimate released sugars by DNS method (Miller, 1959). The percentage of saccharification was calculated by using following formula

Saccharification (%)=Reducing sugar released X 0.9Content of carbohydrate in biomassX 100

## Results

### Xylanase production in submerged fermentation

**Figure 1:**
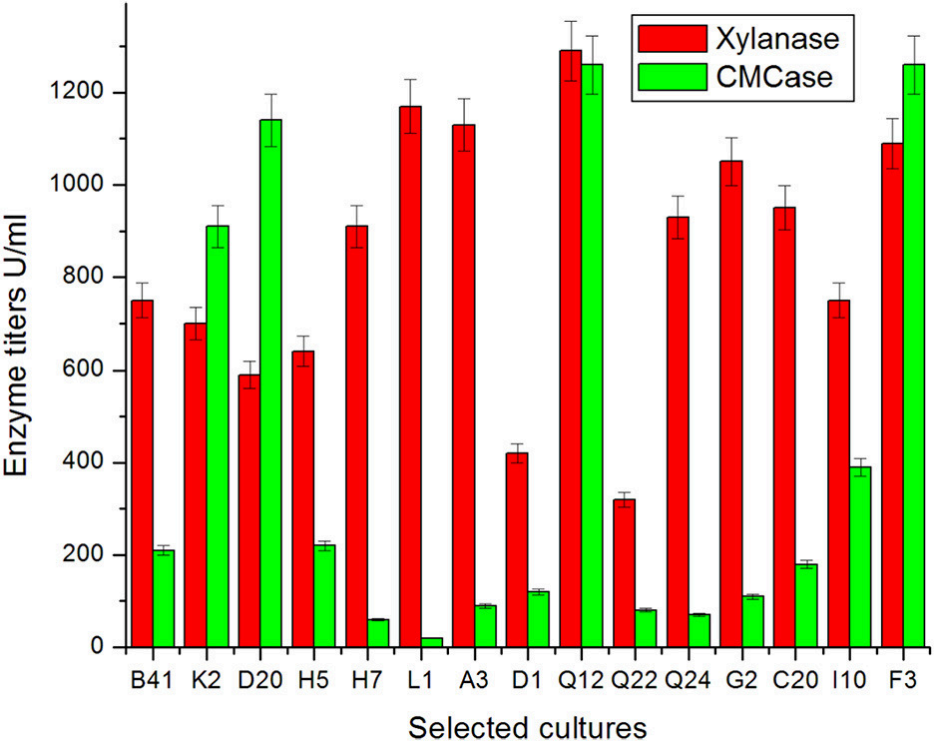
**Secretion of xylanase and CMCase by fungal isolates of different locations in Eastern Ghats on xylose medium**.

Fifteen fungal isolates selected in primary screening (Ramanjaneyulu et al., 2015) were grown in submerged fermentation for 7 days. It was found that all the fungal isolates showed xylanase production within a range of 320–1290 U/ml (Figure 1). Among isolates tested in the present study, some exhibited cellulase-free xylanase activity and some cellulase associated xylanases (Figure 1). The highest xylanase production (1290 U/ml) was observed with an isolate of Q12 followed by L1 (1170 U/ml), A3 (1130 U/ml), and F3 (1090 U/ ml) and the lowest xylanase production was recorded against Q22 (320 U/ml) (Figure 1). The specific activity of xylanase in culture filtrate of these cultures ranged from 356 to 87 U/mg of protein with maximum and minimum recorded in respect of Q12 and Q22, respectively. It was incidental that release of maximum extracellular protein content and maximum biomass was also recorded for highly rated xylanase producing isolates (Table 3). There was not much difference in initial and final pH of the culture broth of all tested isolates in SmF at end of incubation. pH of culture broth of different isolates was recorded in a range of pH 6.04–7.15 (Table 3).

**Table 3:** **Growth, extracellular secretion of protein and change in pH by fungal isolates on xylose medium**.

**S. No**	**Sample**	**Protein mg/ml**	**pH**	**Biomass mg/flask**
1	B41	2.950	7.08	0.090
2	D20	2.625	7.13	0.086
3	K2	2.330	7.06	0.117
4	H5	2.910	7.02	0.155
5	H7	2.845	6.99	0.089
6	L1	3.095	7.12	0.237
7	A3	3.075	7.15	0.150
8	D1	3.295	6.20	0.102
9	Q12	3.620	6.04	0.110
10	Q22	3.695	6.73	0.073
11	Q24	3.215	7.05	0.084
12	G2	3.430	6.58	0.089
13	C20	3.650	6.65	0.103
14	I10	3.550	6.40	0.080
15	F3	3.200	7.13	0.098

### Molecular identification

**Figure 2:**
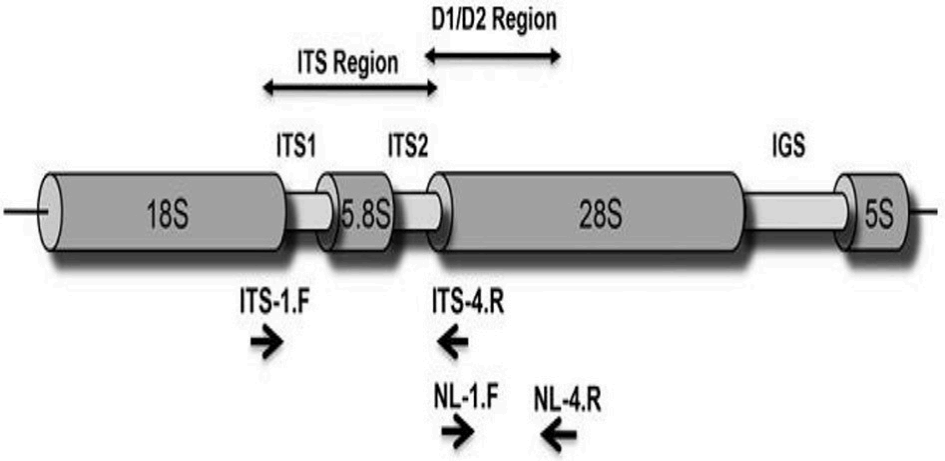
**ITS region of fungi (Romanelli et al., 2014)**.

The ITS region (Figure 2) of rRNA genes in genomic DNA of the potential isolates was analyzed to arrive at identity of the selected potential strains (Romanelli et al., 2014). Different sizes of PCR amplified product (Q12: 466 and L1: 583) in ITS region of rRNA genes were obtained for the two strains. The sequence of PCR product was determined and raw sequences were assembled. The assembled contigs of ITS regions were subjected to NCBI-BLAST database for searching homology with previously existing sequences (Altschul et al., 1990) in Gene Bank to identify the organism based on identity, query coverage, *e*-value, maximum score, and total score. The two isolates showed an identity between 99 and 100% with query coverage of 98–100% and given the names accordingly. The sequences of isolates (Data Sheets 1, 2) and BLAST results (Data Sheets 3, 4) were shown in **Supplementary Data Sheets**.

Furthermore, phylogenetic analysis was carried out with top 25 matches of the sequences by neighbor joining method in *Molecular Evolutionary Genetics Analysis 6.0v* (MEGA 6.0v). The phylogenetic relationships were established by comparing the ITS region of rDNA sequences of the selected strains against the top 25 sequences obtained from NCBI-BLAST database through multiple alignment in clustal W of MEGA 6.0v. Phylogenetic trees were constructed with application of MEGA 6.0 version (trial) by Neighbor-joining method. The phylogenetic trees of Q12 and L1 isolates were shown in Figures (Figures S1, S2).

Sequences of ITS region of rDNA genes of the potential isolates—Q12 and L1 were deposited in NCBI-Gene Bank with KT119615 and KT119619. The isolates Q-12 and L1 are identified as *Fusarium* sp. BVKT R2 and *Fusarium strain* BRR R6, respectively.

### Optimization of xylanase production by response surface methodology (RSM)

In order to optimize the cultivation conditions for xylanase production by the promising *Fusarium* sp. BVKT R2 through RSM, 50 experimental runs in CCD with the combination of five factors—sorbitol, yeast extract (YE), initial pH, temperature, and agitation named *A, B, C, D*, and *E*, respectively were carried out (Table 2). Maximum production of xylanase to the tune of 4560 U/ml in run 2 was recorded as against minimum xylanase production of 320 U/ml in run 24 and 38 in the present study. Thus, it is clear from the results that *Fusarium* sp. BVKT R2 secreted very high yields of xylanase under optimal conditions.

The data obtained from experimental runs in CCD design in the present study fitted into the following second order polynomial equation

$$\begin{array}{l} Y=\beta_{0}+\overset{K}{\underset{i\;=\;1}{\sum }}\beta_{i}X_{i}+\overset{K}{\underset{i\;=\;1}{\sum }}\beta_{ii}X_{i}^{2}+\overset{K-1}{\underset{i\;=\;1,i<j}{\sum }}\overset{K}{\underset{j\;=\;2}{\sum }}\beta_{ij}X_{i}X_{j} \end{array}$$

in which *Y* is the forecasted response; *n* is the number of factors; *x*_*i*_ and *x*_*j*_ are the coded variables; β_0_ is the offset term; β_*i*_, β_*ii*_, and β_*ij*_ are the second-order, quadratic, and interaction effects, respectively; _*i*_ and _*j*_ are the index numbers for factor; and *e*_*i*_ is the residual error and were statistically assessed by ANOVA and multiple regression analysis. Processing of data indicated a model with magnitude of relationship between the response obtained and quantum of variables applied in experimental run.

The final equation in terms of coded values is as follows

$$\begin{array}{ll} Xylanase\;activity\;\left(Y\right)=2662.84+414.12^{*}A-29.12^{*}B\\ +17.06^{*}C-28.68^{*}D+23.09^{*}E\\ -20.16^{*}AB+43.28^{*}AC-26.41^{*}AD\\ +24.53^{*}AE+12.66^{*}BC-32.66^{*}BD\\ +35.78^{*}BE+15.16^{*}CD-17.66^{*}CE\\ +31.41^{*}DE-649.05^{*}A^{\wedge }2\\ -329.05^{*}B^{\wedge }2-284.05*C^{\wedge }2\\ - & 296.55^{*}D^{\wedge }2-271.55^{*}E^{\wedge }2 \end{array}$$

### Interpretation of interaction effects between the independent factors and localization of optimum condition for xylanase production in SmF

**Figure 3:**
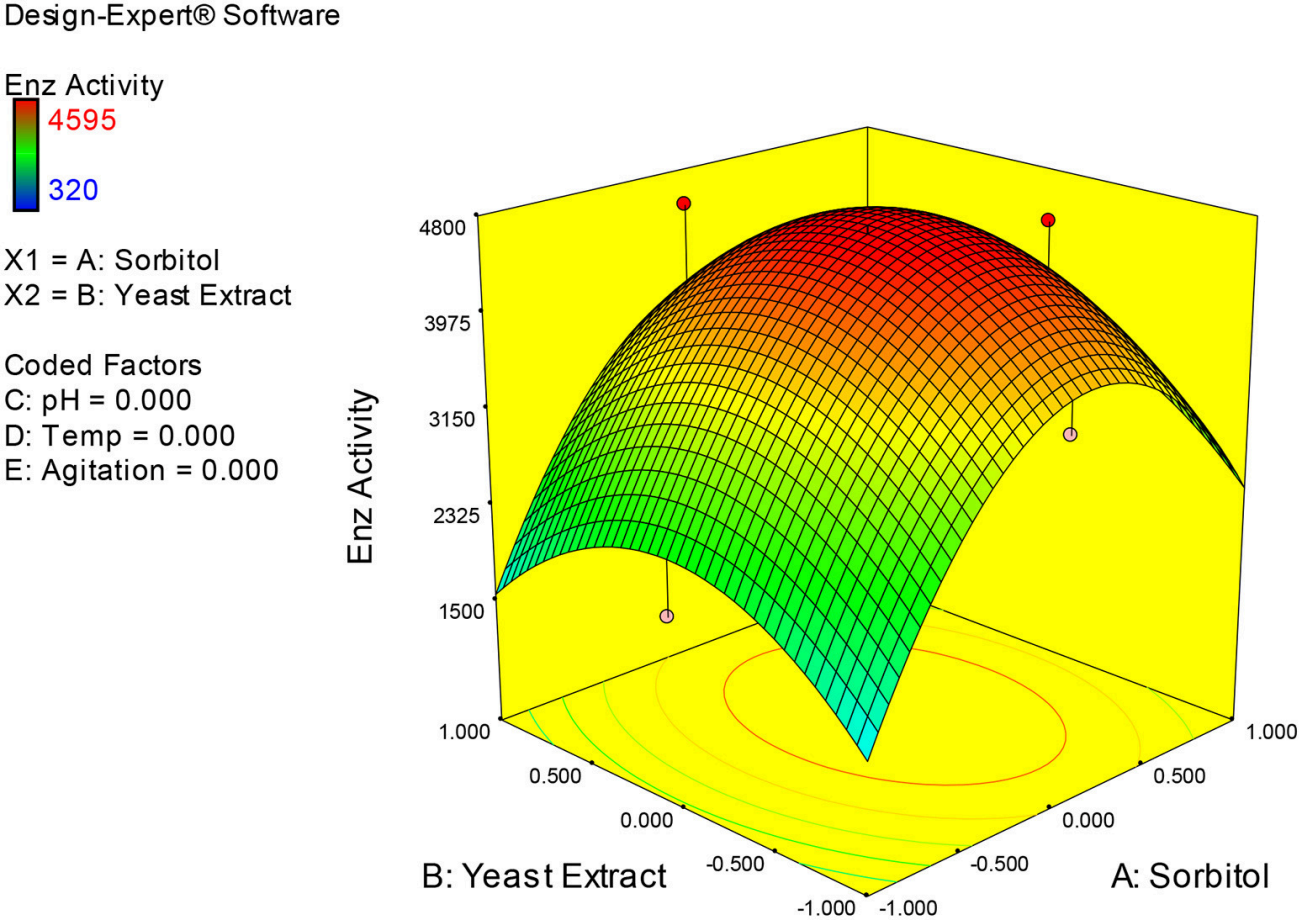
**3D response surface plot showing interaction effects of sorbitol and yeast extract**.

**Figure 4:**
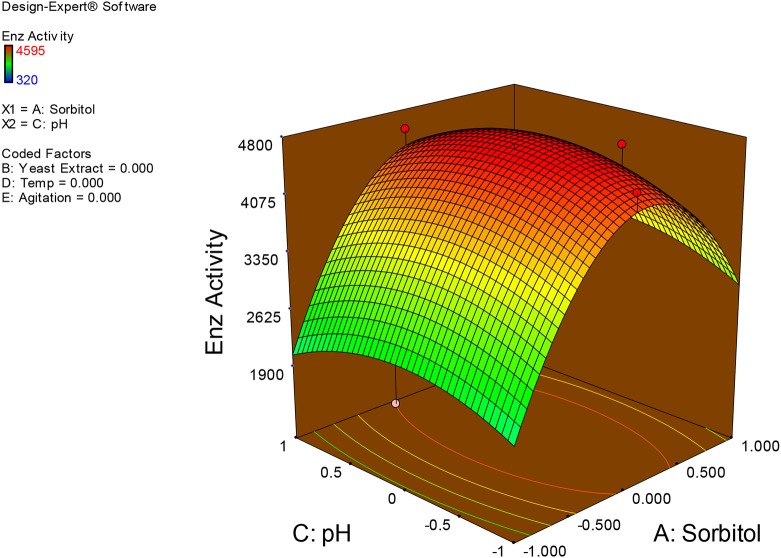
**3D response surface plot showing interaction effects of sorbitol and pH**.

**Figure 5:**
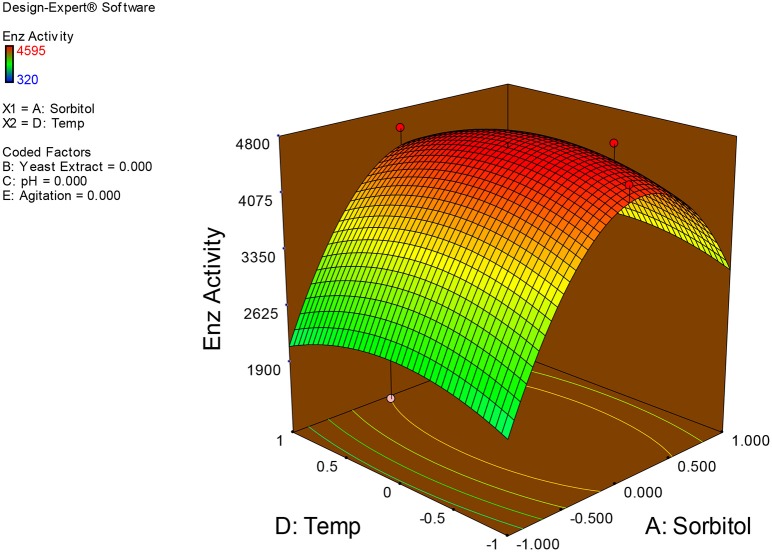
**3D response surface plot showing interaction effects of sorbitol and temperature**.

**Figure 6:**
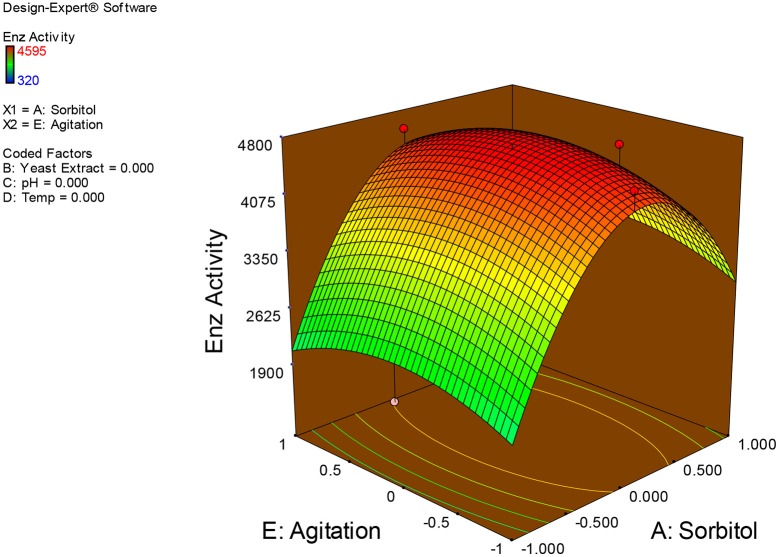
**3D response surface plot showing interaction effects of sorbitol and agitation**.

**Figure 7:**
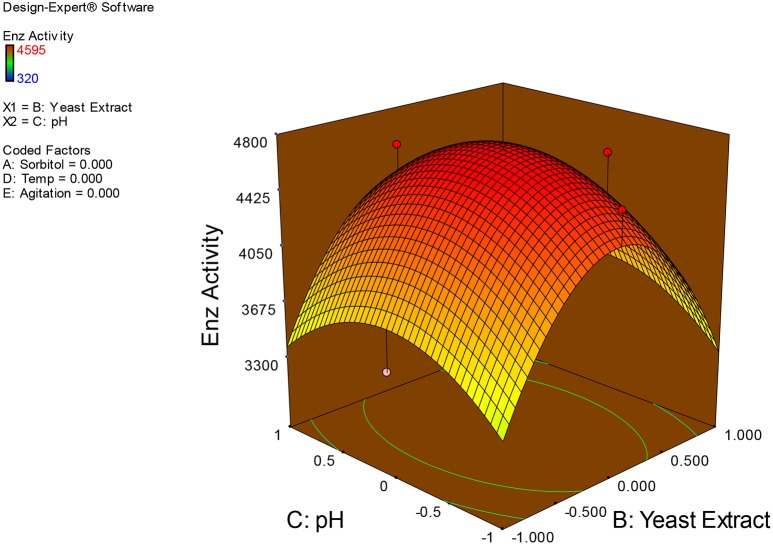
**3D response surface plot showing interaction effects of yeast extract and pH**.

**Figure 8:**
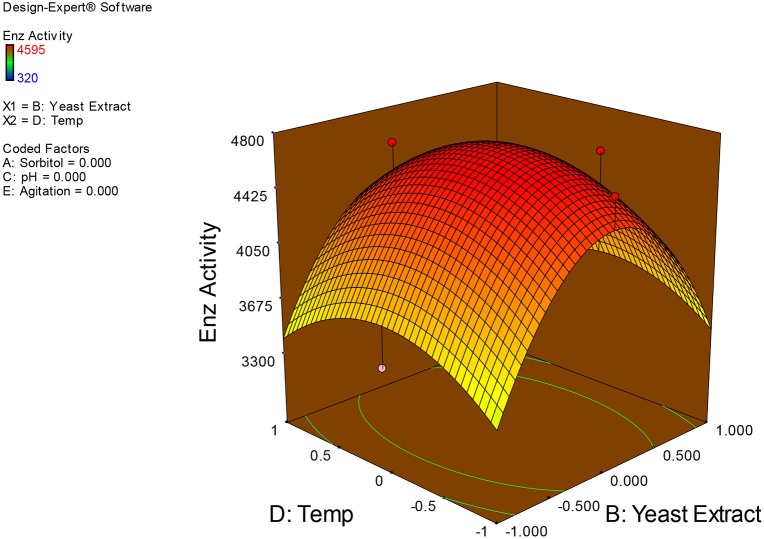
**3D response surface plot showing interaction effects of yeast extract and temperature**.

**Figure 9:**
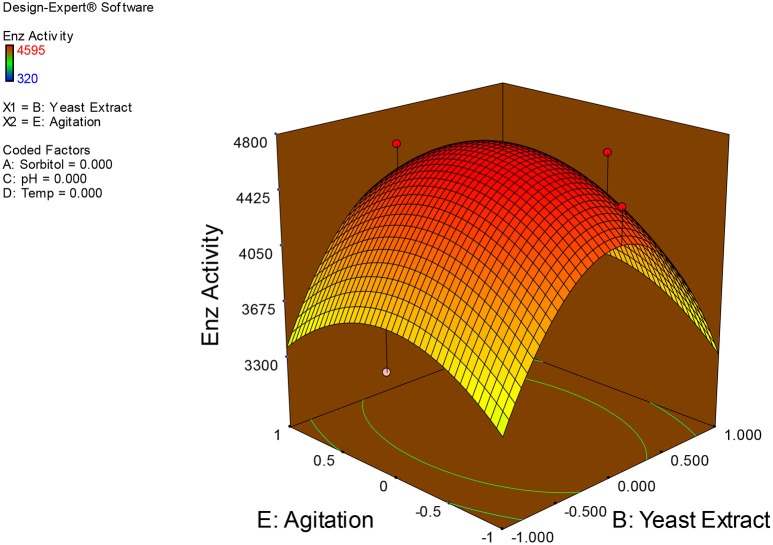
**3D response surface plot showing interaction effects of yeast extract and agitation**.

**Figure 10:**
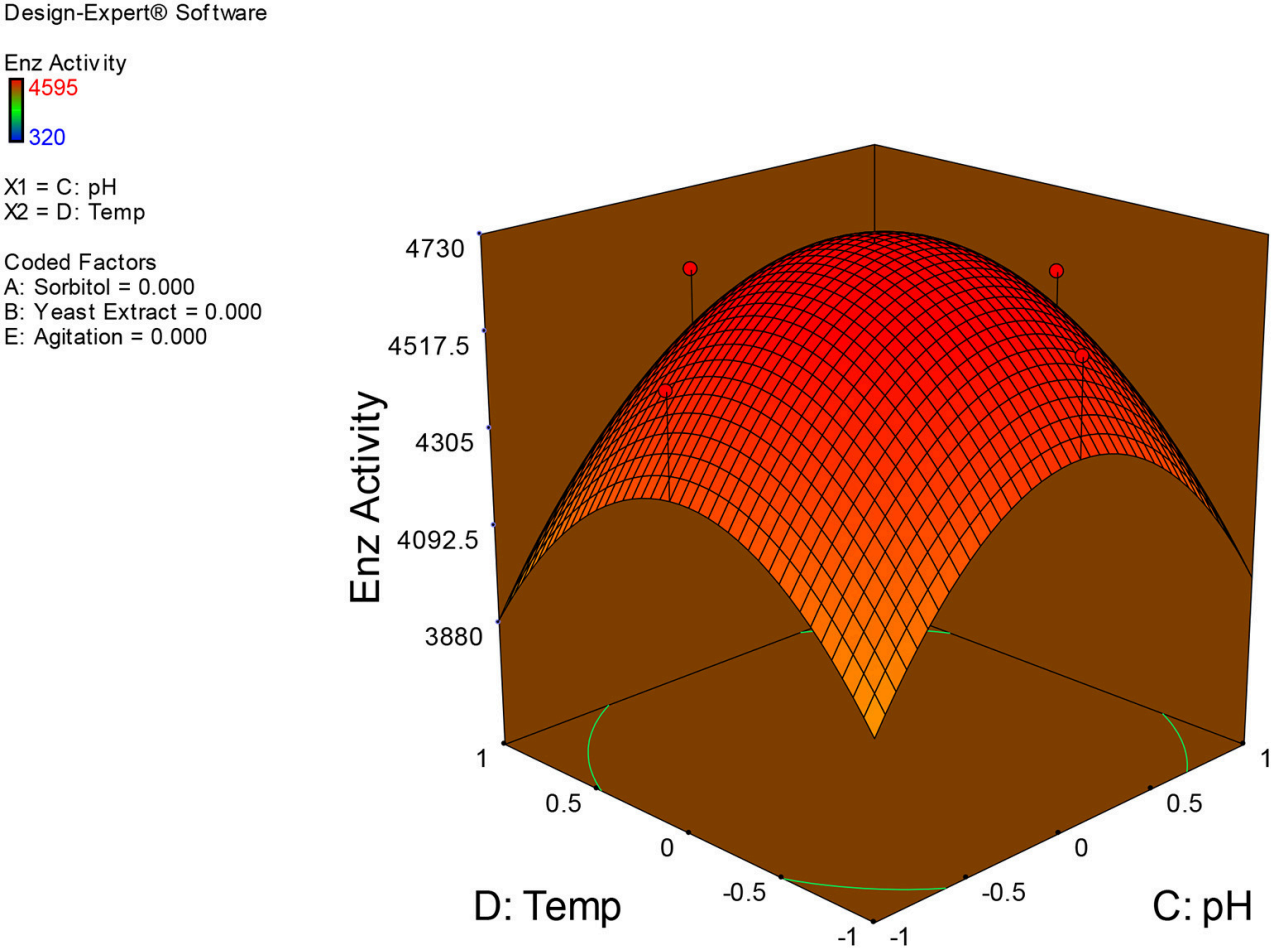
**3D response surface plot showing interaction effects of pH and temperature**.

**Figure 11:**
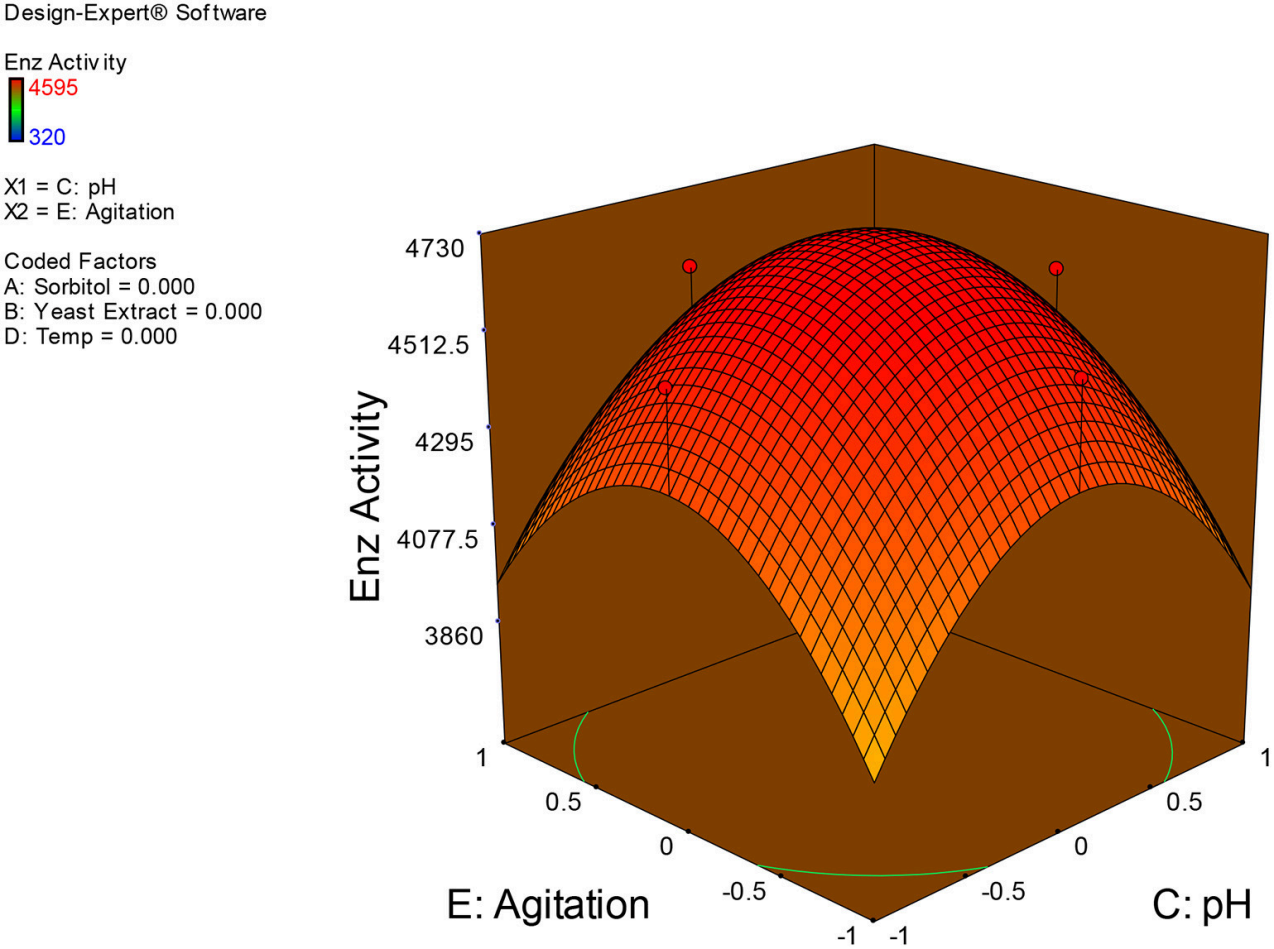
**3D response surface plot showing interaction effects of pH and agitation**.

**Figure 12:**
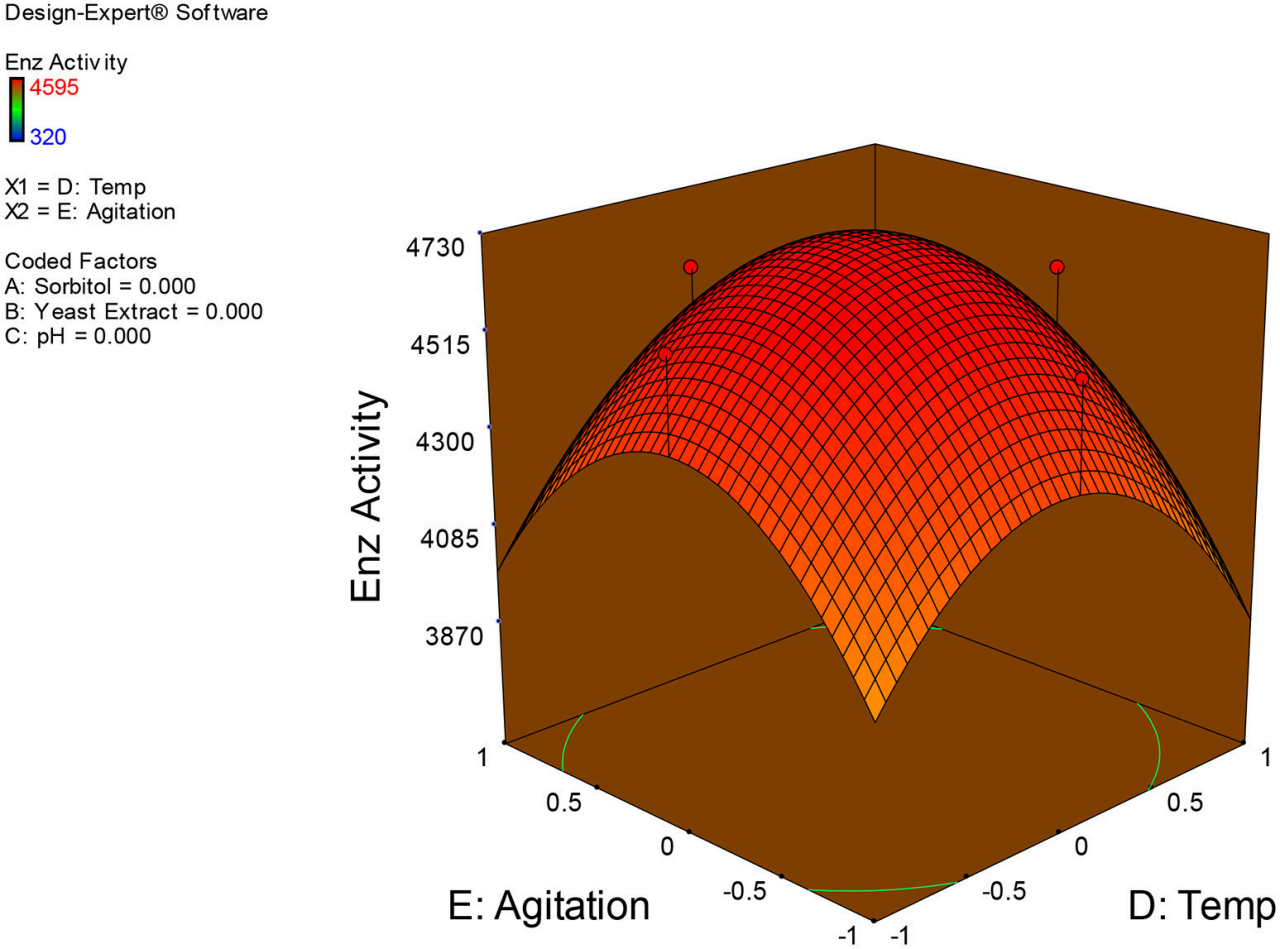
**3D response surface plot showing interaction effects of temperature and agitation**.

Examination of the data on lines of the response surface model with reliance on quadratic polynomial equation permitted us to not only arrive at the optimum culture conditions for growth of the potential *Fusarium* sp. BVKT R2 in the submerged fermentation but also assess interaction effects among variables—sorbitol concentration, yeast extract concentration, pH, temperature, and agitation on xylanase production. Series of response plots (Figures 3–12) described relationships between variables and responses (xylanase yields).

Figures 3–12 show interaction effects between sorbitol and YE (a), sorbitol and pH (b), sorbitol and temperature (c), sorbitol and agitation (d), YE and pH (e), YE and temperature (f), YE and agitation (g), pH and temperature (h), pH and agitation (i) and temperature and agitation (j) and optimal culture conditions for the production of xylanase by *Fusarium* sp. BVKT R2 observed in interaction studies are sorbitol 1.5%, YE 1.5%, pH 5.0, temperature 32.5 and agitation of 175 rpm with maximum xylanase yield of 4560 U/ml.

### Saccharification of lignocellulosic biomass

**Figure 13:**
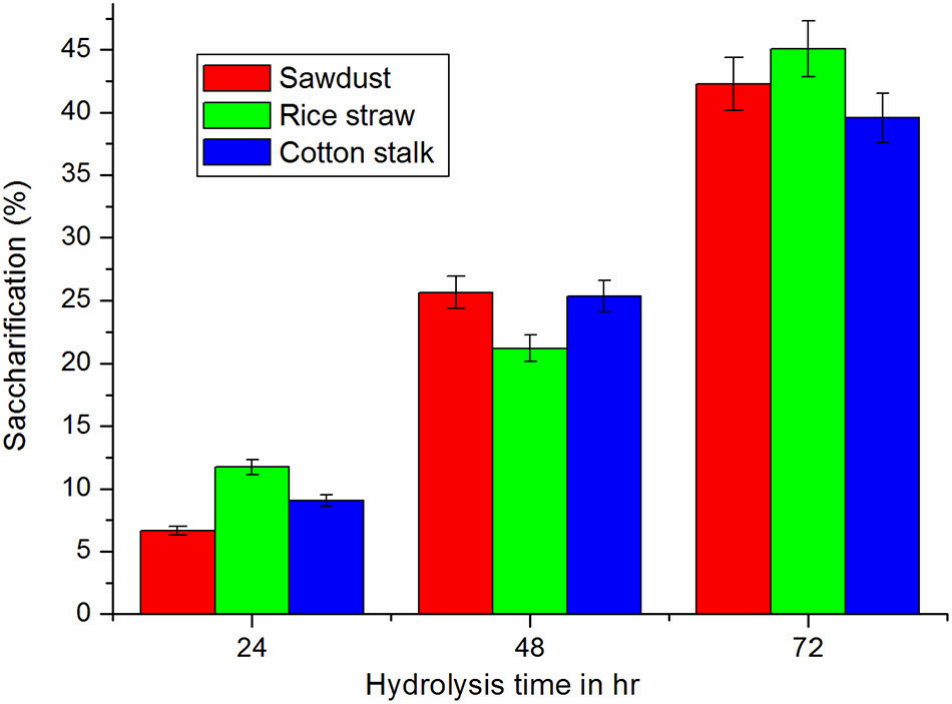
**Saccharification (%) of biomass with crude enzyme of ***Fusarium*** sp. BVKT R2**.

Saccharification is the most important step in biorefinary of lignocellulosic biomass. Hence, release of sugars from untreated lignocellulosic wastes—sawdust, rice straw, and cotton stalk were tested with culture filtrate of *Fusarium* sp. BVKT R2 (crude enzyme) at 37°C for saccharification. The maximum amount of reducing sugars with the highest saccharification of 45.07% was liberated from rice straw followed by sawdust (42.28%) and cotton stalk (39.56%) at the end of 72 h of incubation time (Figure 13). The least saccharification was observed in case of sawdust (6.67%) after 24 h incubation. The release of reducing sugars was increased with increase in the incubation time. The results clearly indicate that xylanase of *Fusarium* sp. BVKT R2 has the potential to saccharify all the lignocellulosic biomasses tested. The saccharification percentage varied from 6.67 to 45.07 among the substrates at different tested time intervals.

## Discussion

It is clear from the results of the present study that fungal isolates—L1, Q12, F3, and A3 were rated high for xylanase production. Similarly, Abdel-Satera and El-Said (2001) (Dereeper et al., 2008) screened xylan-degrading filamentous fungi and reported that *Trichoderma harzianum* produced maximum xylanase production when grown at incubation temperature of 35°C. Isolation of fungal cultures with xylanase activity was made from different sources such as active compost, decaying organic materials, and soil samples (Saha and Ghosh, 2014; Adhyaru et al., 2015; Pereira et al., 2015). Senthil et al. (2005), Abdullah et al. (2014), and Bekkarevich et al. (2015) reported xylanase activity of fungal and bacterial cultures within a range of 182–3060 U.

In this study, two fungal species were identified on ITS sequencing analysis—Q12 isolate as *Fusarium* sp. BVKT R2 and L1 isolate as *Fusarium* strain BRR R6. Sequencing of ITS region is reliable and is commonly used for the identification of fungi including pathogenic one (Romanelli et al., 2010; Fuerst et al., 2015). ITS region of rDNA sequences is considered as DNA barcode for the identification of fungi (Bellemain et al., 2010). Chen et al. (2001), Iwen et al. (2002), Buee et al. (2009), Jumpponen and Jones (2009), Jumpponen et al. (2010), Romanelli et al. (2010), and Ghannoum et al. (2010) pointed that the ITS region in a broad spectrum of fungi can be amplified and can generally be recovered in a single PCR amplicon, since the amplification is usually 400–700 bp in length. Similarly, PCR amplification and sequencing of ITS region facilitated not only to identify pathogenic fungi and members of fungal communities in environmental samples but also establish a phylogenetic relationship among them (Borman et al., 2008; Nilsson et al., 2009). ITS sequence of fungal organisms, deposited into international sequence database—NCBI, serves as reference for identification of fungal isolates made from different habitats (Nilsson et al., 2009).

Prediction of operation of variables for production of xylanase by *Fusarium* sp. BVRK2 in SmF in a quadratic model can be made with a variety of criteria. According to application of ANOVA (Table 4) the estimated coefficient of determination *R*^2^ (0.9406) was in close confirmity with the adjusted *R*^2^ (0.9697) indicating significance of the model. The significance of model is also evident from the model *F*-value of 79.36 (Table 4). There is only 0.01% probability for occurrence of large model *F*-value due to noise. Regression models with *R*^2^-value greater than 0.9 and closer to 1.0 indicates good correlation between experimental and predicted values (Haaland, 1989). The coefficient of variation (CV) indicates the degree of precision. Lower value of coefficient of variation (4.83%) in the present study indicated a better precision and reliability of experiments. Adequate precision measures the signal to noise ratio and a ratio greater than 4 is desirable. Our model ratio of 22.749 indicates an adequate signal. Hence, the model can be used to navigate the design space. Values of “Prob > *F*” less than 0.05 indicate that model terms are significant. In this study the significant model terms are A, B, C, D, AB, AC, AD, BC, BD, CD, CE, A^2^, B^2^, and C^2^.

**Table 4:** **ANOVA for response surface quadratic model**.

**Source**	**Sum of squares**	***Df***	**Mean squares**	***F*-value**	***p*-value Prob > *F***
Model	1.511E+008	20	7.554E+006	79.36	<0.0001^*^
A-Sorbitol	7.605E+006	1	7.605E+006	79.90	<0.0001^*^
B-Yeast extract	2.94	1	2.94	3.090E−005	0.0327^**^
C-pH	9894.12	1	9894.12	0.10	0.0495^**^
D-Temp	33,988.97	1	33,988.97	0.36	0.0448^**^
E-Agitation	18,124.26	1	18,124.26	0.19	0.0658
AB	13,000.78	1	13,000.78	0.14	0.0144^*^
AC	59,944.53	1	59,944.53	0.63	0.0339^**^
AD	22,313.28	1	22,313.28	0.23	0.0319^**^
AE	19,257.03	1	19,257.03	0.20	0.6562
BC	5125.78	1	5125.78	0.054	0.0181^**^
BD	34,125.78	1	34,125.78	0.36	0.0540^**^
BE	40,969.53	1	40,969.53	0.43	0.5169
CD	7350.78	1	7350.78	0.077	0.0031^**^
CE	9975.78	1	9975.78	0.10	0.0485^**^
DE	31,563.28	1	31,563.28	0.33	0.0691
A2	7.753E+006	1	7.753E+006	81.45	<0.0001^*^
B2	2.234E+006	1	2.234E+006	23.47	<0.0001^*^
C2	4.066E+005	1	4.066E+005	4.27	0.0478^**^
D2	3.348E+005	1	3.348E+005	3.52	0.0708
E2	3.819E+005	1	3.819E+005	4.01	0.0546^**^
Residual	2.760E+006	29	95179.23		
Lack of fit	2.741E+006	22	1.246E+005		
Pure error	19362.88	7	2766.13		
Core total	1.538E+008	49			

*R^2^, 0.9821; Adj. R^2^, 0.9697; Pred. R^2^, 0.9406; C.V., 4.83%; Adeq. Precision, 22.749*.

** p < 0.05,

* *p < 0.01*.

The plot of natural logarithm (In) of the residual SS (Sum of Squares) against λ suddenly dropped with a minimum value in the region of the best optimum value 0.88 (Figure S3). As the current value of confidence interval with λ was very close to the optimum value, the data did not need a transformation (Box and Cox, 1964). The minimal and maximal values of confidence interval were 0.58 and 1.22 respectively according to the model (Figure S3). As another plot of residuals proceeded along a straight line with satisfaction of normal assumption (Figure S4), inference was drawn that the model could satisfactorily describe xylanase production by RSM (Myers and Montgomery, 2002).

**Figure 14:**
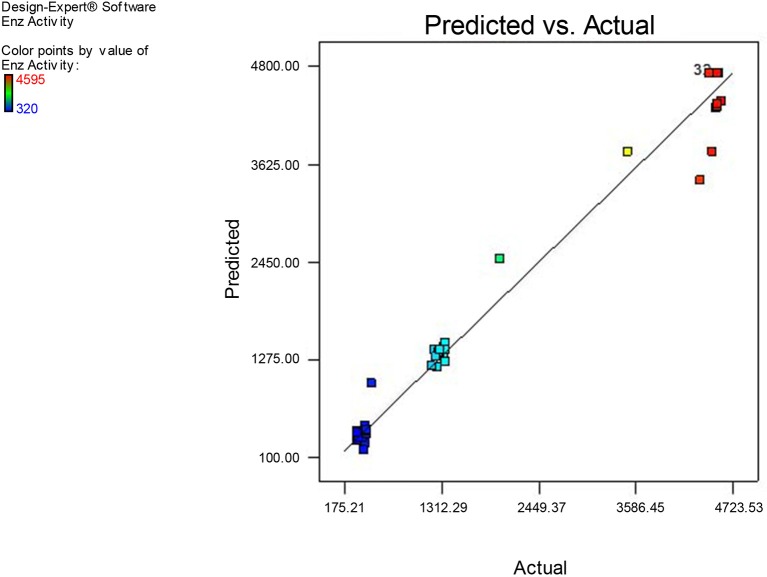
**Actual experimental and predicted values of xylanase production**.

Comparison of observed and predicted xylanase production by using the second-order regression analysis, the model predicted the response (xylanase activity) related to particular values of the regressor variables. The plot for the observed xylanase activity (the response) vs. model predicted xylanase activity demonstrates that these are very close with each other. As can be found that the observed xylanase activity (response) and model predicted xylanase activity, data points are split by 45° line implying a reasonable agreement of the predicted response with the observed ones. The result from three replications was correspondence with the predicted value and the model was proven to be adequate. The good correlation between predicted and experimental values after optimization justified the model validity. Figure 14 demonstrates that the experimental xylanase activity values are in well agreement with predicted response values.

To get maximum yield of xylanase production point prediction was used to assess the model validity and to assess optimal level parameters. It was predicted that at the specified optimum conditions (Sorbitol 1.5%, YE 1.5%, pH: 5.0, Temperature: 32.5°C, and agitation of 175 rpm) the response of xylanase was 4560 U/ml. Validation studies were performed in triplicate for further confirmation as suggested by the model. The outcome of optimized process conditions yielded the xylanase production of 4560 U/ml. The experimental value (4548 U/ml) was very closer to the predicted value (4560 U/ml). It was clear from these results demonstrated that the model was useful to predict the xylanase yield as well as the optimization of experimental fermentation conditions. Hence, the CCD based RSM models appeared to be accurate and reliable for predicting the production of xylanase by *Fusarium* sp. BVKT R2. Similarly, optimization of growth conditions in respect of *Bacillus circulans* (Heck et al., 2005) and *Schizophyllum commune* (Haltrich et al., 1993) through statistical methods resulted in improvement of xylanase yields by three-folds over un-optimized conditions and yields obtained were close to predicted values. Optimization of influential parameters—carbon source, nitrogen source and incubation temperature for *Aspergillus candidus* enhanced xylanase level to 70 U/ml in 48 h (Garai and Kumar, 2013).

The viability of lignocellulosic biofuel industry will depend on development of cheaper and compatible enzyme cocktail for release of sugars from saccharification of biomass feedstock (Boonyuen et al., 2014). In this study, both xylanase and cellulase enzyme present cultural filtrate of *Fusarium* sp. BVKT R2 were able to saccharify untreated lignocellulosic biomass to the tune of 47%. The ability of crude enzyme of different organisms to saccharify lignocellulosic biomass was evaluated by different researchers (Harshvardhan et al., 2013; Santhi et al., 2014; Premalatha et al., 2015). Saccharification of pretreated sugarcane bagasse with crude cellulase enzyme of *Fomitopsis* sp. RCK 201 at loading of 20 U/g released 1.5–2.4-fold higher sugars than in the case of untreated sugar cane bagasse (Deswal et al., 2014). Crude cellulase enzyme of *Aspergillus niger* could release sugars from pretreated sawdust at higher rate than from untreated sawdust (Sridevi et al., 2015). Saccharification occured to the extent of 45–84% in pretreated paddy straw and sorghum with enzyme extract of seaweed-associated cellulolytic bacterial cultures (Santhi et al., 2014). Among different plant biomasses tested with cellulolytic enzymes of *Enhydrobacter* sp. ACCA2, maximum saccharification (61.33%) was observed for bamboo on third day of incubation (Premalatha et al., 2015). Saccharification of macro-algae *Ulva lactuca* biomass with cellulolytic enzyme from a marine *bacillus* sp. released increased recovery of glucose of 450 mg/g (Harshvardhan et al., 2013). In-depth studies on saccharification of untreated/treated lignocellulosic biomass with crude enzyme of *Fusarium* sp. BVKT R2 in comparison with commercial enzymes under different conditions will further assess saccharification potential.

## Conclusions

In this study, xylanase producing fungal strains were isolated from forest soil samples and two potential strains were identified based on ITS gene sequencing. The Isolate *Fusarium* sp. BVKT R2 (Q12) was optimized for both physical and nutrient parameters through RSM and yielded xylanase as high as 4560 U/ ml in SmF under optimal conditions of 1.5% sorbitol, 1.5% yeast extract, pH of 5.0, temperature of 32.5°C and agitation of 175 rpm. The results indicate that the model developed for optimal production of xylanase by isolate *Fusarium* sp. is reliable and accurate. Crude xylanase of *Fusarium* sp. BVKT R2 could achieve release of sugars (45%) from untreated biomasses in saccharification process indicating that plant cell wall polysaccharide hydrolyzing enzyme from the potential fungal culture has potential for saccharification process in conversion of lignocelluloses to value-added products. Thus, forest soil samples serve as a massive treasure-house of xylan-utilizing microflora for exploration for industrial applications.

## Author contributions

Both authors—GR and BR made equal contribution toward the paper. The results presented in the manuscript are a part of doctoral programme of GR. GR conducted experiments and collected results from different experiments and critically analyzed them and finally presented them in the manuscript form. BR acted as supervisor for GR in doctoral programme and is involved at every stage—designing of experiments, generation of ideas, and analysis of results and overall improvement of manuscript.

### Conflict of interest statement

The authors declare that the research was conducted in the absence of any commercial or financial relationships that could be construed as a potential conflict of interest.

## Supplementary material

Data Sheet 1 **Nucleotide sequence of ITS region of fungal isolate Q12**.

Data Sheet 2 **Nucleotide sequence of ITS region of fungal isolate L1**.

Data Sheet 3 **Multiple sequence alignment of ITS region of fungal isolate Q12**.

Data Sheet 4 **Multiple sequence alignment of ITS region of fungal isolate L1**.

Figure S1 **Phylogenetic tree of Q12 isolate (***Fungus s***p. BVKT R2)**.

Figure S2 **Phylogenetic tree of L1 isolate (***Fusarium*** sp. BRR R6)**.

Figure S3 **Box-Cox plot for power transforms**.

Figure S4 **Plot of internally studentized residuals vs. normal percentage probability**.
