# Supplementary material for: Optimization of Xylanase Production through Response Surface Methodology by Fusarium sp. BVKT R2 Isolated from Forest Soil and Its Application in Saccharification
Source: Front Microbiol. 2016 Sep 22;7:1450. doi: 10.3389/fmicb.2016.01450 (PMC5032753; doi:10.3389/fmicb.2016.01450)
Supplement: Data Sheet 1 — Nucleotide sequence of ITS region of fungal isolate Q12. [file DataSheet1.DOCX]

**Nucleotide Sequence of Isolate Q 12**

GAGGGATCATTACCGAGTCTTCCAACTCCCAAAACCCCTGTGAACATACCAATTGTTGCCTCGGCGGATCAGCCCGCTCCCGGTAAAACGGGACGGCCCGCCAGAGGACCCCTAAACTCTGTTTCTATATGTAACTTCTGAGTAAAACCATAAATAAATCAAAACTTTCAACAACGGATCTCTTGGTTCTGGCATCGATGAAGAACGCAGCAAAATGCGATAAGTAATGTGAATTGCAGAATTCAGTGAATCATCGAATCTTTGAACGCACATTGCGCCCGCCAGTATTCTGGCGGGCATGCCTGTTCGAGCGTCATTTCAACCCTCAAGCCCCCGGGTTTGGTGTTGGGGATCGGCGAGCCCTTGCGGCAAGCCGGCCCCGAAATCTAGTGGCGGTCTCGCTGCAGCTTCCATTGCGTAGTAGTAAAACCCTCGCAACTGGTACGCGGCGCGGCCAAGCCGTTAA
