# Supplementary material for: Optimization of Xylanase Production through Response Surface Methodology by Fusarium sp. BVKT R2 Isolated from Forest Soil and Its Application in Saccharification
Source: Front Microbiol. 2016 Sep 22;7:1450. doi: 10.3389/fmicb.2016.01450 (PMC5032753; doi:10.3389/fmicb.2016.01450)
Supplement: Data Sheet 3 — Multiple sequence alignment of ITS region of fungal isolate Q12. [file DataSheet3.docx]

**Data sheet 3. Multiple sequence alignment of ITS region of fungal isolate Q12.**

| S. No. | Organism | Max. score | Total score | Query coverage (%) | E value | Identity (%) | Accession No. |
| --- | --- | --- | --- | --- | --- | --- | --- |
| 1. | *Fusarium sp. fSS-2015* | 843 | 843 | 100 | 0.0 | 99 | FJ847730 |
| 2. | *Fusarium sp. FZ0802* | 843 | 843 | 100 | 0.0 | 99 | KJ765860 |
| 3. | *Fusarium proliferatum Fp 1114M* | 843 | 843 | 100 | 0.0 | 99 | KP670435 |
| 4. | *Fusarium proliferatum CBS 189.38* | 843 | 843 | 100 | 0.0 | 99 | KM231816 |
| 5. | *Fusarium proliferatum CBS 263.54* | 843 | 843 | 100 | 0.0 | 99 | KM231815 |
| 6. | *Fusarium verticillioides MPS13* | 843 | 843 | 100 | 0.0 | 99 | KP760060 |
| 7. | *Fusarium sp. MPS12* | 843 | 843 | 100 | 0.0 | 99 | KP760059 |
| 8. | *Fusarium proliferatum haplotype F405* | 843 | 843 | 100 | 0.0 | 99 | KJ435281 |
| 9. | *Fusarium sp. HR7* | 843 | 843 | 100 | 0.0 | 99 | KP091294 |
| 10. | *Fusarium proliferatum FS20* | 843 | 843 | 100 | 0.0 | 99 | KP689223 |
| 11. | *Fusarium proliferatum ZB074* | 843 | 843 | 100 | 0.0 | 99 | KJ528883 |
| 12. | *Fusarium proliferatum PT5* | 843 | 843 | 100 | 0.0 | 99 | KP313248 |
| 13. | *Fusarium verticillioides voucher RIFA 187* | 843 | 843 | 100 | 0.0 | 99 | KF624791 |
| 14. | *Fusarium oxysporum KDF-11* | 843 | 843 | 100 | 0.0 | 99 | KC963036 |
| 15. | *Fusarium sp. BAB-4364* | 843 | 843 | 100 | 0.0 | 99 | KM401408 |
