# Supplementary material for: Optimization of Xylanase Production through Response Surface Methodology by Fusarium sp. BVKT R2 Isolated from Forest Soil and Its Application in Saccharification
Source: Front Microbiol. 2016 Sep 22;7:1450. doi: 10.3389/fmicb.2016.01450 (PMC5032753; doi:10.3389/fmicb.2016.01450)
Supplement: Data Sheet 4 — Multiple sequence alignment of ITS region of fungal isolate L1. [file DataSheet4.docx]

**Data sheet 4. Multiple sequence alignment of ITS region of fungal isolate L1.**

| S. No. | Organism | Max. score | Total score | Query (%) | E value | Identity (%) | Accession No. |
| --- | --- | --- | --- | --- | --- | --- | --- |
| 1. | *Fusarium proliferatum* | 1059 | 1059 | 100 | 0.0 | 99 | HF930594 |
| 2. | *Fusarium sp.*G39 | 1057 | 1057 | 99 | 0.0 | 99 | JO623494 |
| 3. | *Fusarium proliferatum* M14 | 1053 | 1053 | 99 | 0.0 | 99 | KP132230 |
| 4. | *Fusarium fujikuroi* IMI 58289 | 1053 | 1053 | 99 | 0.0 | 99 | HF679024 |
| 5. | *Fusarium oxysporum* KAMIL01 | 1053 | 1053 | 99 | 0.0 | 99 | KC119203 |
| 6. | *Fusarium sp*. KANPR01 | 1053 | 1053 | 99 | 0.0 | 99 | KC119197 |
| 7. | *Gibberella fujikuroi* | 1053 | 1053 | 99 | 0.0 | 99 | AB237662 |
| 8. | Gibberella moniliformis YLJ-57 | 1051 | 1051 | 99 | 0.0 | 99 | JX231007 |
| 9. | Gibberella sp. ZTMS-2011 | 1051 | 1051 | 99 | 0.0 | 99 | HO630966 |
| 10. | Fungal sp. ARIZL424 | 1051 | 1051 | 99 | 0.0 | 99 | FJ512808 |
| 11. | *Sordaromycete sp*. 7670B | 1051 | 1051 | 99 | 0.0 | 99 | EU680539 |
| 12. | *Gibberella moniliformis* strain Fm-X.1.7-030527-31 | 1051 | 1051 | 99 | 0.0 | 99 | EU364864 |
| 13. | *Fusarium proliferatum* NRRL 31071 | 1050 | 1050 | 99 | 0.0 | 99 | AF291061 |
| 14. | *Gibberella moniliformis* | 1050 | 1050 | 99 | 0.0 | 99 | AB369908 |
| 15. | Fungal sp. ARIZ L329 | 1050 | 1050 | 99 | 0.0 | 99 | FJ512780 |
