# Supplementary figures and images for: Optimization of Xylanase Production through Response Surface Methodology by Fusarium sp. BVKT R2 Isolated from Forest Soil and Its Application in Saccharification
Source: Front Microbiol. 2016 Sep 22;7:1450. doi: 10.3389/fmicb.2016.01450 (PMC5032753; doi:10.3389/fmicb.2016.01450)

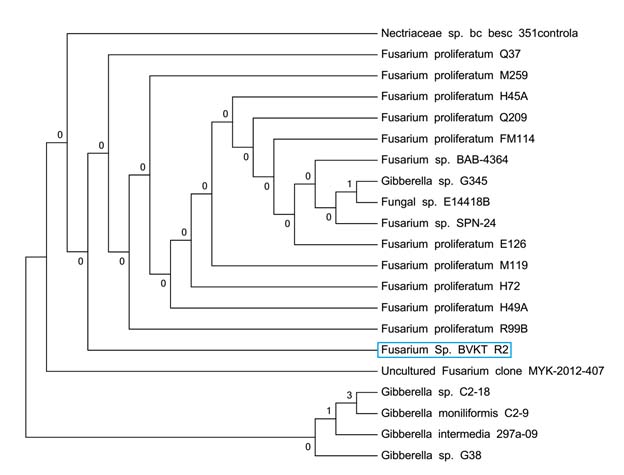

Supplement: Figure S1 — Phylogenetic tree of Q12 isolate (Fungus sp. BVKT R2). [file Image1.JPEG]

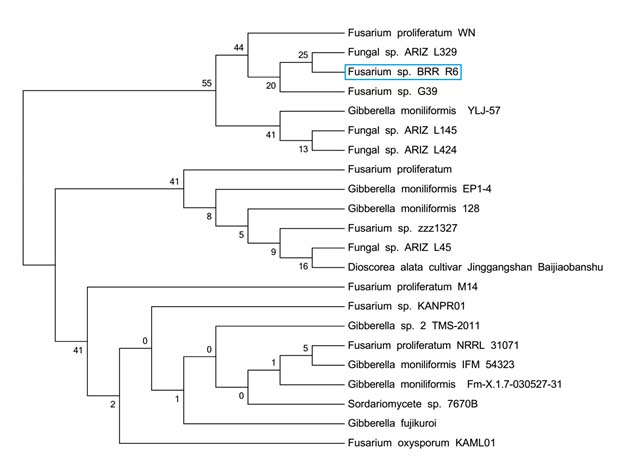

Supplement: Figure S2 — Phylogenetic tree of L1 isolate (Fusarium sp. BRR R6). [file Image2.JPEG]

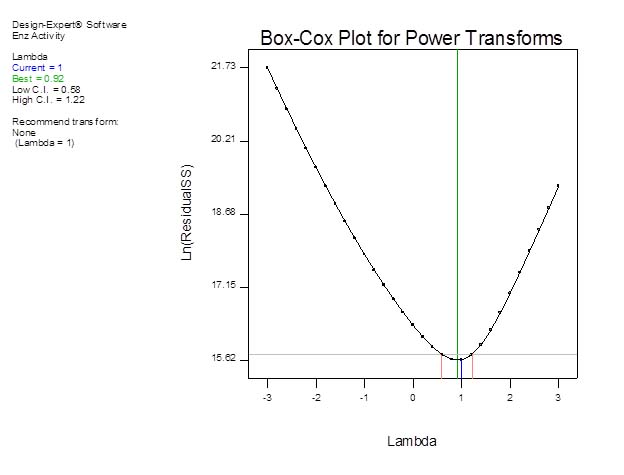

Supplement: Figure S3 — Box-Cox plot for power transforms. [file Image3.JPEG]

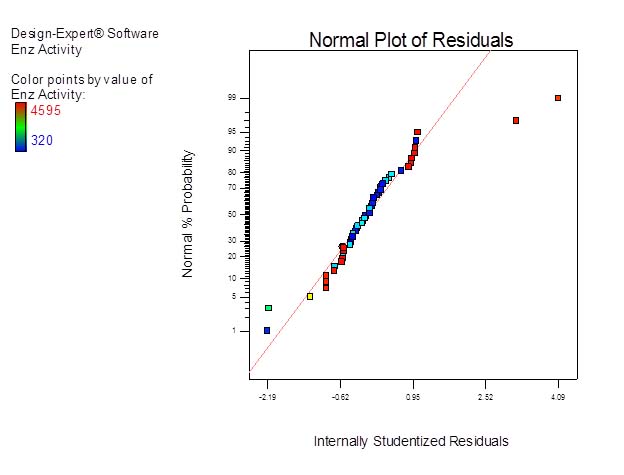

Supplement: Figure S4 — Plot of internally studentized residuals vs. normal percentage probability. [file Image4.JPEG]
